# CT of the medial clavicular epiphysis for forensic age estimation: hands up?

**DOI:** 10.1007/s00414-021-02516-z

**Published:** 2021-02-24

**Authors:** Magdalini Tozakidou, Rieke L. Meister, Lennart Well, Kay U. Petersen, Sebastian Schindera, Eilin Jopp-van Well, Klaus Püschel, Jochen Herrmann

**Affiliations:** 1 Section of Pediatric Radiology, Department of Diagnostic and Interventional Radiology and Nuclear Medicine, University Medical Center Hamburg-Eppendorf, Martinistraße 52, 20246 Hamburg, Germany; 2 Department of Diagnostic and Interventional Radiology and Nuclear Medicine, University Medical Center Hamburg-Eppendorf, Hamburg, Germany; 3 Department of Psychiatry and Psychotherapy, University Hospital Tübingen, Tübingen, Germany; 4 Department of Radiology, Cantonal Hospital Aarau, Aarau, Switzerland; 5 Department of Forensic Medicine, University Medical Center Hamburg-Eppendorf, Hamburg, Germany

**Keywords:** Image noise, CT, Forensic age estimation, Patient positioning

## Abstract

**Purpose:**

The aim of this study was to assess the impact of arm position in computed tomography (CT) of the clavicle performed for forensic age estimation on clavicular position, image noise, and radiation dose.

**Methods and materials:**

Forty-seven CT scans of the medial clavicular epiphysis performed for forensic age estimation were conducted with either hands and arms held upwards (CT_HU,_ 28 persons) or positioned at the body (CT_HD_, 19 persons). Presets were identical for both positions (70 mAs/140 kVp; Brilliance iCT, Philips). Each CT scan was reconstructed with an iterative algorithm (i-Dose 4) and evaluated at the middle of the sternoclavicular joint. Clavicular angle was measured on a.p. topograms in relation to a horizontal line. Quantitative image noise was measured in air at the level of medial clavicular epiphysis. Effective dose and scan length were recorded.

**Results:**

Hands-up position compared with hands-down position resulted in a lower lateral body diameter (CT_HU_ 41.1 ± 3.6 cm vs. CT_HD_ 44.6 ± 3.1 cm; *P* = 0.03), a reduced quantitative image noise (CT_HU_: 39.5 ± 9.2; CT_HD_: 46.2 ± 8.3; *P* = 0.02), and lower CTDI_vol_ (5.1 ± 1.4 mGy vs. 6.7 ± 1.8 mGy; *P* = 0.001). Scan length was longer in patients examined with hands up (HU: 8.5 ± 3.4 cm; HD: 6.2 ± 2.1 cm; *P* = 0.006). Mean effective dose for CT_HU_ was 0.79 ± 0.32 mSv compared with 0.95 ± 0.38 mSv in CT_HD_ (*P* = 0.12). Clavicular angle was 17° ± 6° in patients with hands down and 32° ± 7° in patients with hands up (*P* < 0.001).

**Conclusion:**

By elevated arm positioning, the image quality of clavicular CT scans can be improved while maintaining radiation dose compared with hands down. Clavicular position differs according to the hand position. Thus, positioning patients with elevated hands is advisable for forensic clavicular CT examinations, but multiplanar CT reconstructions should be adjusted to clavicular position and scan length should be reduced to a minimum.

## Introduction

With increasing migration into European countries, forensic age assessment in young adults has become increasingly important [1]. When the chronological age cannot be determined alone by non-medical means, medical imaging may be added to specify an age range [2, 3]. Provided that x-ray examinations are legally permitted, the AGFAD (Arbeitsgemeinschaft für Forensische Altersdiagnostik, Study Group on Forensic Age Diagnostics) recommends a steps-wise approach beginning with a conventional x-ray examination of the left hand or the teeth [2]. A computed tomography (CT) of the medial clavicle may be supplemented if a minimal age > 18 or > 21 years needs to be clarified [3–5]. Minimizing radiation dose remains an important part of imaging, especially since clavicular CT is often performed in young and borderline adults due to the greater risk of malignancy after radiation exposure [6].

Dose reduction strategies may include automatic tube current modulation, different ways of mathematical postprocessing algorithms, such as iterative reconstructions, but also protocol adjustment to patient habitus and proper patient positioning [7–9]. For example, in cervical spine, CT examinations with a low shoulder position allow the application of lower radiation dose with no reduction of image quality [10]. Likewise, in trauma patients, arm-raising for thoracoabdominal CT results in higher image quality and lower radiation dose [7].

To our knowledge, no definite guidelines on patient positioning for forensic clavicular CT imaging have been published so far. Yet, it is common practice to use an arms-down position in forensic age estimation clavicular CT protocols because the clavicular shaft better aligns with the standard axial imaging planes. A number of studies recently suggested to use thin-sliced CT with a multiplanar reformation algorithm which would also allow for a more variable arm positioning [11–15].

The aim of this study was to assess the impact of hand position in clavicular CT performed for forensic age estimation on image noise, image reconstruction, and radiation dose.

## Materials and methods

### Patients and CT protocol

Institutional review board approval was waived for this retrospective investigation. CT examinations during the period 10/2017 to 10/2018 from 28 male persons (mean age 20.5 ± 3.4 years as reported by the subjects) were included. Results were compared with data from a previously published collective [16], which had been examined with hands positioned next to the body (hands down, CT_HD_).

**Fig. 1:**
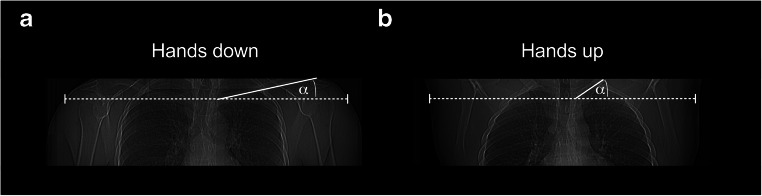
Coronal topograms of example patients positioned with hands lateral to the thorax (**a**) and with hands up (**b**). Lateral diameter was measured at the level of the medial clavicular epiphysis (dotted line). Clavicular angle (*α*) was measured between the line through the medial half of the clavicle and a horizontal line

A 256-multidetector CT scanner (Brilliance iCT, Philips, the Netherlands) was used to perform unenhanced CT scans of the clavicle. Scan parameters are summarized in Table 1. Subjects were in a supine position and asked to place their hands, arms, and shoulders upwards (CT_HU_). The anteroposterior (ap) distance and lateral body diameter at the midlevel of the medial clavicular epiphysis was measured in subjects, in which the entire soft tissues were included in the scan. Lateral diameter was available in eight subjects with hands down and in 27 patients with hands up, anteroposterior diameter in 12 subjects with hands down and in 24 subjects with hands up. Scan length was documented in all patients. The clavicular angle was measured on ap topograms in relation to a horizontal line (Fig. 1).

### Image noise

Image noise was measured in air at the midlevel of medial clavicular epiphysis by one pediatric radiologist with 8 years of experience in CT imaging as described earlier [16]. The standard deviation of mean CT numbers measured in Hounsfield units (HU) within a ROI was documented in three ROIs on each side.

### Radiation exposure

The CT automatically generated the volume CT dose index (CTDI_vol_) and the dose–length product (DLP). In addition, we calculated a theoretical DLP for a normalized scan length of 5 cm, which was thought to be sufficient for scanning the medial clavicular epiphysis. For estimation of the effective dose the DLP was multiplied by an organ specific conversion coefficient of 0.0137 mSv/mGycm for an adult chest region at 140 kVp as described previously [16, 17].

### Image reconstruction

**Fig. 2:**
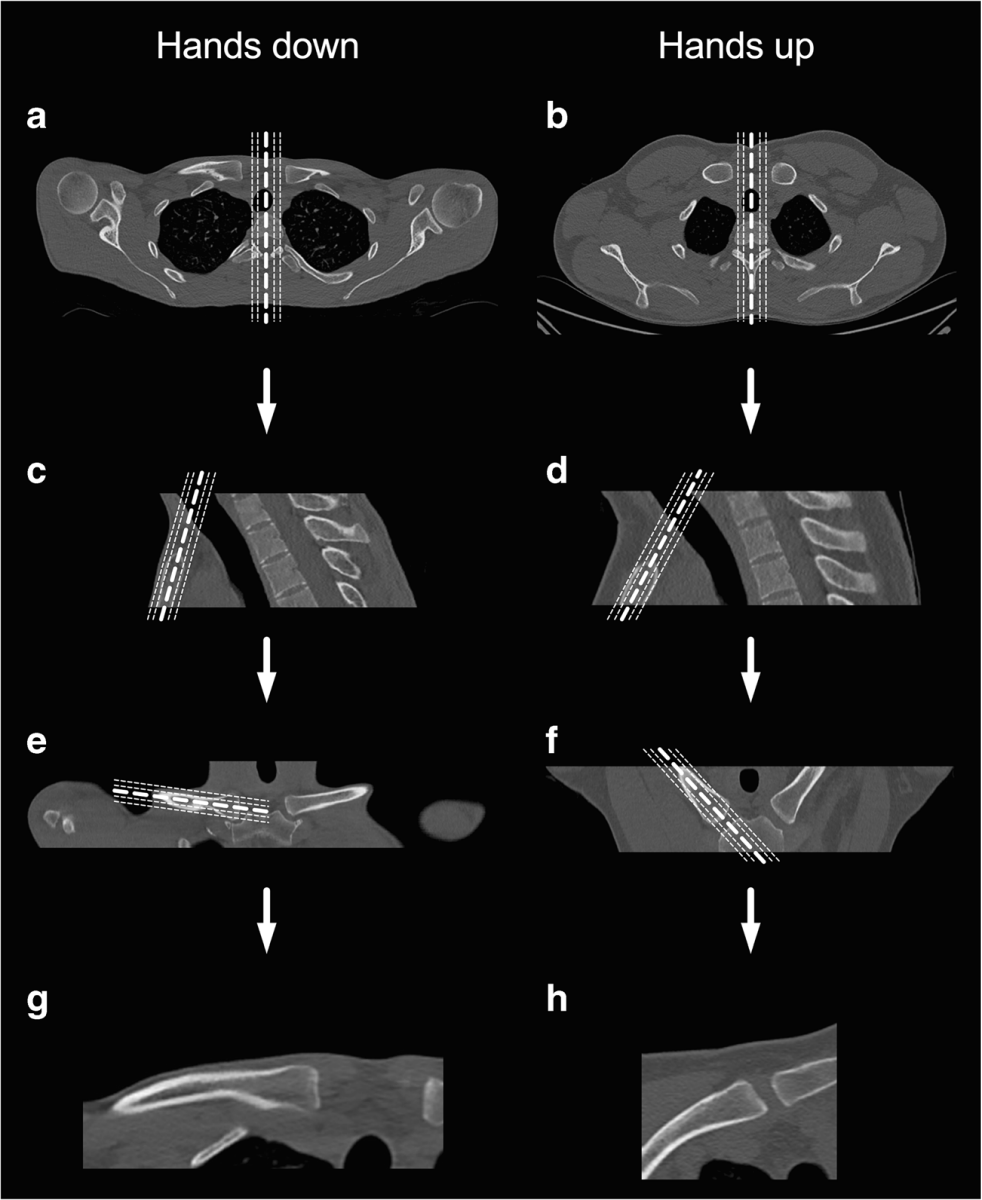
Systematic reformations of the medial clavicular epiphysis reconstructed with iDose 4 using bone convolution kernel (window level/width, 500/2500). Starting from axial planes (**a**, **b**) and sagittal reformations were performed (**c**, **d**). Coronal images were aligned along the sternum (**e**, **f**). On coronal images, each clavicle was reformatted along the clavicular shaft separately for each side, resulting in adapted axial slices. Example reformations of the right side are shown in **g** and **h**

Since differences of classifications in axial and coronal reformations and influence of slice thickness have been described [12, 13], images were reviewed using multiplanar reformations (MPRs) with slice thickness/increment of 1.0/0.5 mm on a PACS workstation. In order to avoid anatomic distortion due to changes in clavicular position in subjects with hands up, reformations were aligned along the sternum for coronal views and along the clavicular shaft for adapted axial views in all scans (Fig. 2).

### Statistical analysis

To examine possible correlations of scan length and radiation dose, the nonparametric Spearman correlation coefficient has been calculated. For the comparison of image noise between subjects with arms up and subjects with arms down, the mean values of six measurements in each subjects were used and Student’s *t* tests were performed. Statistical tests were performed by using appropriate statistical software (SPSS Statistics, version 25 and GraphPad QuickCalcs). A *P* value < 0.05 was considered statistically significant.

**Table 1:** Scan and reconstruction parameters

Parameter	Value
Tube voltage [cm]	140 kVp
Reference tube-current-time product	70 mAs
Rotation time	0.5 sec
Pitch factor	0.579
Detector configuration	64 × 0.625 mm
Automatic tube-current modulation	Z-Dom (Philips Healthcare)
Image reconstruction algorithm	iDose 4 (Philips Healthcare), bone convolution kernel [18] *
Window level/width	500/2500
Section thickness/increment	1.0/0.5 mm
Field-of-view	15 × 15 cm to 20 × 20 cm, depending on the person’s constitution

## Results

A total of 28 CT scans of the medial clavicles with hands up and 19 CT with hands down were evaluated. The anthropomorphic parameters were available in 23 patients with hands up and in 19 patients with hands down and did not differ between both groups (body weight: HD: 68 ± 8 kg vs HU 68 ± 8 kg, *P* = 0.81; height: HD 174 ± 5 cm vs HU 175 ± 8 cm; *P* = 0.65).

### Body diameter, clavicular angle in relation to arm posture, and multiplanar reformations

The lateral body diameter was less with hands positioned upward than downward (CT_HU_ 41.1 ± 3.6 cm vs. CT_HD_ 44.6 ± 3.1 cm; *P* = 0.03). The ap diameter was not significantly different between both groups (CT_HU_ 14.7 ± 0.9 cm vs. CT_HD_ 14.4 ± 1.4 cm; *P* = 0.38). The clavicular angle increased significantly with hands positioned upward compared with arms positioned downward (right side, 32° ± 7° vs, 18° ± 6°; left side, 31° ± 7° vs. 17°± 6°; *P* < 0.001; Fig. 1). Example reformations in patients with hands up and hands down are shown in Fig. 2. While the orientation of adapted axial images in patients with hands down is close to axial images of the thorax, the orientation of adapted axial images in patients with hands up differs according the increased clavicular angle (Figs. 1 and 2).

### Image quality and noise

**Fig. 3:**
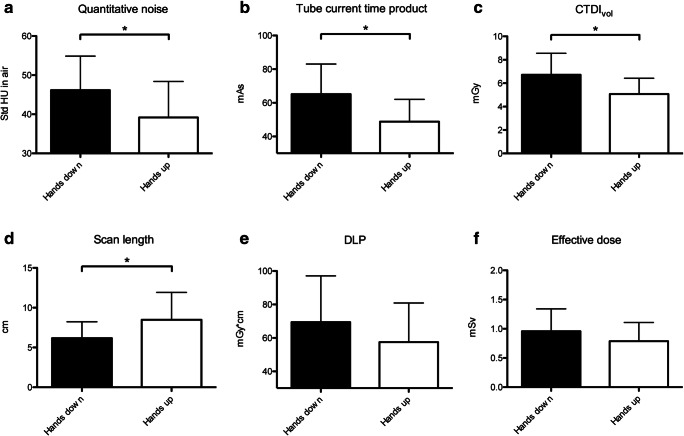
Quantitative image noise (**a**) was evaluated at midlevel of the medial clavicular epiphysis on images reconstructed with iDose 4. Image noise was significantly higher in subjects with hands down than in subjects with hands up. And tube current time product (**b**) and CTDI_vol_ (**c**) was higher in subjects with hands down. Scan length (**d**) was larger in subjects with hands up. DLP (**e**) and effective dose (**f**) showed no significant difference between the two groups. Data are presented as mean ± standard deviation. Asterisks indicate significant differences (*P* < 0.05)

The subjective image quality was diagnostic in all examinations. Quantitative measurements were performed in the bone window and displayed lower image noise in subjects with hands up than in those with hands down (HU: 39.5 ± 9.2; HD: 46.2 ± 8.3; *P* = 0.02; Fig. 3a).

### Radiation exposure

Upward hand position compared with downward hand position resulted in a lower mean effective tube current time product (48.8 ± 12.2 mAs vs. 65.1 ± 17.9 mAs; *P* = 0.001; Fig. 3b) and a lower mean CTDI_vol_ (5.1 ± 1.4 mGy vs. 6.7 ± 1.8 mGy; *P* = 0.001; Fig. 3c). As the mean effective scan length was slightly longer for the hands up than for the hands down position (8.5 ± 3.4 cm vs. 6.2 ± 2.1 cm, *P* = 0.006; Fig. 3d, no significant differences were noted for DLP (57.5 ± 23.4 mGy*cm vs. 69.5 ± 27.6 mGy*cm, *P* = 0.12; Fig. 3e) and for the calculated effective dose (0.79 ± 0.32 mSv vs. 0.95 ± 0.38 mSv, *P* = 0.12; Fig. 3f). When CT examinations were standardized to a theoretical scan length of 5 cm, hands-up position resulted in lower DLP and lower effective dose (25.4 ± 6.7 mGy*cm vs. 33.6 ± 9.2 mGy*cm, ED 0.46 ± 0.13 mSv vs. 0.35 ± 0.09 mSv, *P* = 0.001).

Clavicular angle of both sides showed a moderate positive correlation with scan length (right: Spearman coefficient: 0.37, *P* = 0.01; left: Spearman coefficient: 0.37, *P* = 0.01) and a moderate negative correlation with lateral diameter (right: Spearman coefficient: − 0.597, *P* < 0.001; left: Spearman coefficient: − 0.62, *P* < 0.001).

## Discussion

This work investigated the impact of arm position on depiction of the medial clavicular joint and related imaging parameters including noise as well as radiation dose in forensic clavicular CT examinations. To the best of our best knowledge, no literature has been published on optimal clavicular position in forensic CT examinations.

Several studies have shown that the arm position is relevant for image quality in CT examinations of different body parts. In concordance, we were able to demonstrate a lower image noise in CT examinations with arms up which was attributed to a reduced body diameter in this position. The finding is in line with several previous CT studies showing that upward arm positioning for thoracoabdominal and downward positioning of the shoulders for CT scans of the cervical spine enhance image quality [7, 10].

The aforementioned studies also demonstrated that with proper positioning, a reduction of radiation dose can be reached. Also, in our study, there was a tendency towards lower DLP and effective dose with hand position up which did not reach statistical significance. In fact, the longer scan range in the HU group counteracted the dose saving potentials. When corrected to a scan length of 5 cm, the CTDI_vol_, estimated effective dose, and the effective tube current time product were lower in subjects with elevated hands, which means that at the examined level, the applied dose was less.

Interestingly, we found a positive correlation between clavicular angle and scan length with a longer scan length in those subjects with hands placed upwards. This might be caused by the fact that sometimes, technicians were unsure about the relevant part of the clavicle. When the clavicle is placed horizontally in a small scan field, a major part of the clavicle can still be scanned. This is different in patients with a steep angle of the clavicle, where a longer and often not necessary scan field was applied. Optimal positioning and minimizing the scan length to the relevant part of the medial clavicular epiphysis seems crucial for further dose reduction. Thus, technicians should be trained to position patients properly and to identify the relevant part of the clavicle.

We suggest the use of multiplanar reformations, in which axial views are aligned along the clavicular shaft in order to correct for changes of the clavicular position due to the elevated arms. MPRs are common practice when evaluating images that are acquired with multiple detector CTs [19]. This is especially important when imaging body parts that change their position according to the patient’s body constitution or position in the scanner. Changes of reformation techniques have been reported to influence image quality [13] and viewing images in axial and coronal reformations are considered gold standard for the evaluation of the medial clavicular epiphysis in forensic age estimation [20–22]. While many important studies in the field forensic clavicular imaging have examined patients with hands positioned at the body [12], studies with retrospective analysis of the clavicle, especially those that use thoracic CT examinations, evaluated multiplanar reformations in patients with hands positioned upwards [23]. Furthermore, volume rendering techniques have been discussed for the evaluation of the medial clavicular epiphysis [24]. The adapted reformation of the clavicle and possible also additional use of volume rendering techniques might facilitate the evaluation of the clavicle independent of the patient’s position.

## Limitations

Our study has several limitations. First, we only included 28 subjects with elevated arms, since those were the ones available during the included time period. Second, we only measured noise in the air. As described previously [16], this might not be representative of noise in bone.

## Conclusion

Quantitative image noise in clavicular CT is less when the scan is acquired with an upward arm position. This might facilitate image analysis and open the door for further dose reduction in CT examinations of the medial clavicular epiphysis.

## Declarations

This retrospective study was approved by the local ethics committee with waived informed consent due to the use of anonymized data.
